# Retraction Note: Shoulder range of motion and strength in beach tennis athletes compared to volleyball and tennis players: implications for injury risk and performance

**DOI:** 10.1186/s13018-025-06295-w

**Published:** 2025-09-22

**Authors:** Ana Paula Ramos, Leonardo Luiz Barretti Secchi, Filippo Migliorini, Nicola Maffulli, Rodrigo Okubo

**Affiliations:** 1 Physical Therapy Graduate Program, State University of Santa Catarina, Florianópolis, SC Brazil; 2 Faculdade de Ciências Sociais e Agrárias de Itapeva (FAIT), Itapeva, SP Brazil; 3 Department of Orthopedics and Trauma Surgery, Academic Hospital of Bolzano (SA-BES-ASDAA), Via Lorenz Böhler 5, 39100 Bolzano, Italy; 4 Department of Life Sciences, Health, and Health Professions, Link Campus University, 00165 Rome, Italy; 5 Department of Medicine and Psychology, University La Sapienza, Rome, Italy; 6 School of Pharmacy and Bioengineering, Faculty of Medicine, Keele University, ST4 7QB Stoke on Trent, England; 7 Centre for Sports and Exercise Medicine, Barts and the London School of Medicine and Dentistry, Mile End Hospital, Queen Mary University of London, London, E1 4DG England

**Retraction Note to: Journal of Orthopaedic Surgery and Research (2025) 20:411**. 10.1186/s13018-025-05600-x

The authors have retracted this article. Following the publication of this article, concerns regarding ethics approval were raised. The authors have noted that the cited ethics approval (approval opinion 4.313.311 from the ethics committee of the Federal University of Santa Catarina (UFSC)) lacks authorisation and communication of a specific scope, particularly related to biomechanical assessment procedures reported in this article.

All authors agree with this retraction.

